# Supplementary material for: 3D‐Printing of Electroconductive MXene‐Based Micro‐Meshes in a Biomimetic Hyaluronic Acid‐Based Scaffold Directs and Enhances Electrical Stimulation for Neural Repair Applications
Source: Adv Sci (Weinh). 2025 Jul 15;12(34):e03454. doi: 10.1002/advs.202503454 (PMC12442685; doi:10.1002/advs.202503454)
Supplement: Supplementary file 1 — Supporting Information [file ADVS-12-e03454-s001.docx]

**Supplementary Figures:**

**
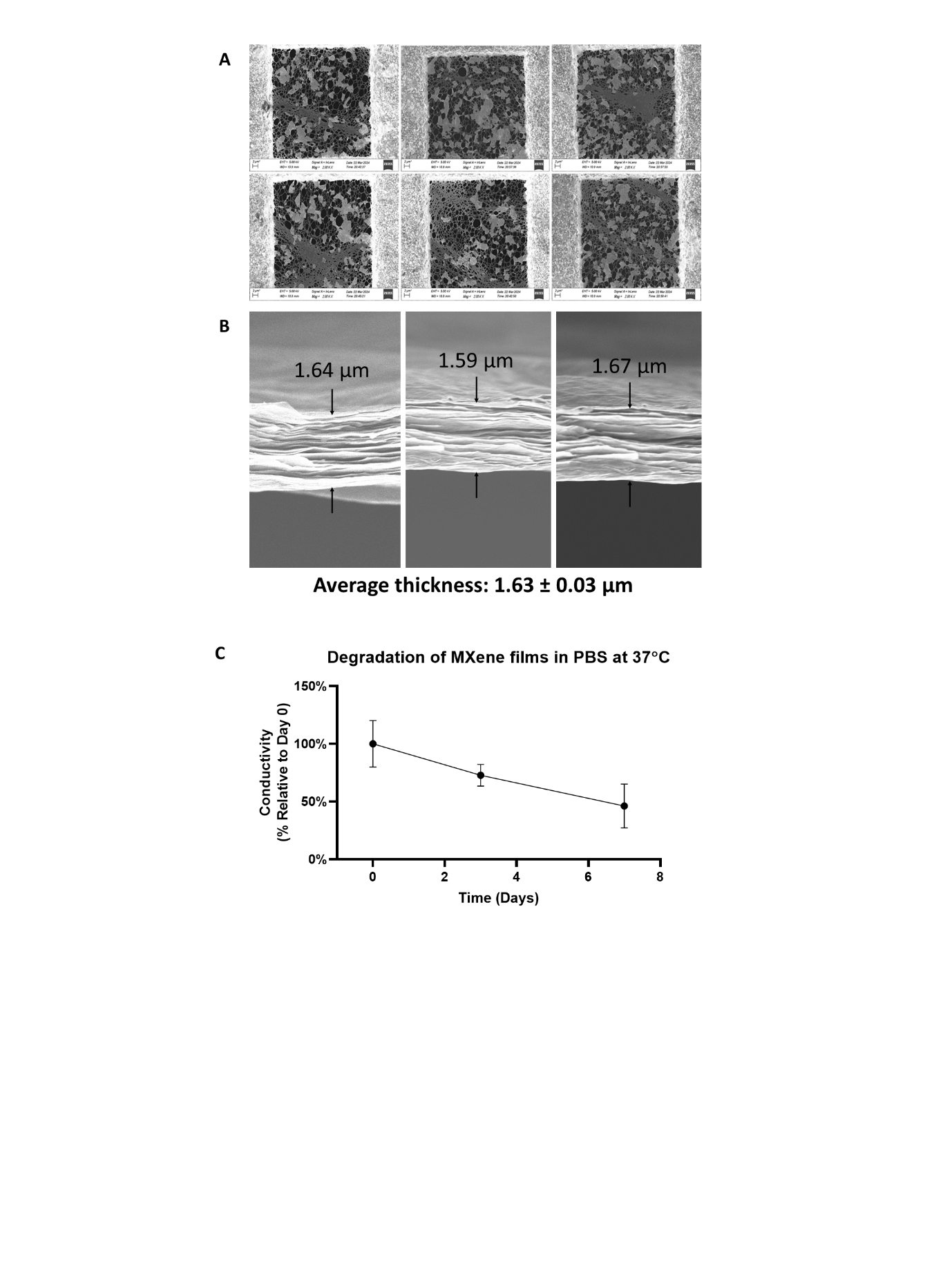
**

**Supplementary Figure S1.** MXene Characterization Data. (A) Size distribution of MXene nanosheets. (B) Cross-section of MXene films. (C) Degradation of electrical conductivity in ionic medium. MXenes exhibit robust degradation resistance over time in PBS salt solution mimicking the in vivo environment. MXene conductivity decreased to approximately 46.2 ± 19.1% after 7 days of degradation.


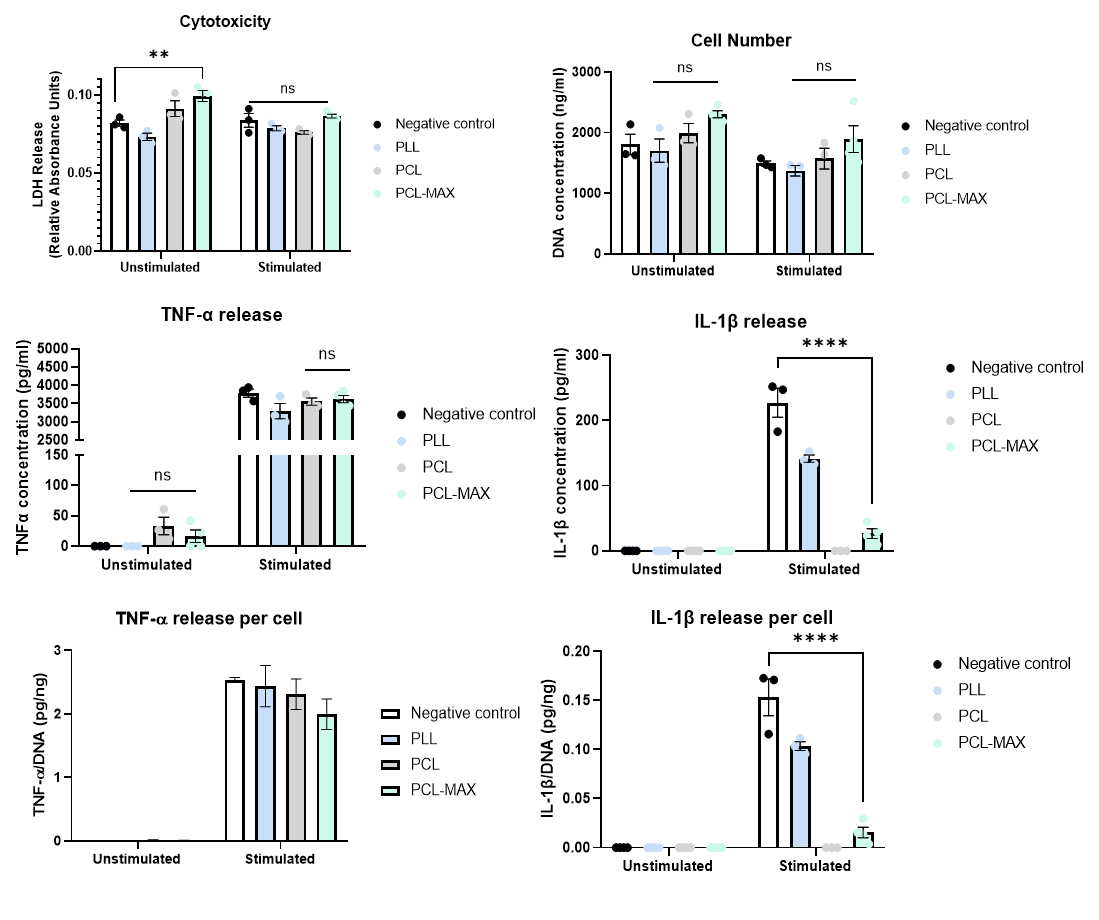


**Supplementary Figure S2. Microglial Compatability Analysis.** (A) Analysis of LPS release indicates a small but significant increase in LDH release from unstimulated microglia on MXene/PCL substratres but significant difference compared to PCL-only substrates. No difference was observed across stimulated microglial cultures. (B) No change in cell number was observed between substrates. (C) No significant increase in TNF-α release was observed. PCL/MXene substrates exhibited significantly lower IL-1β release from stimulated microglia compared to well-plate surfaces or PLL-coated substrates. (E) No significant changes in TNF-α release were observed on a per-cell basis. (F) PCL/MXene substrates exhibited significantly decreased release of IL-1β release on a per-cell basis compared to well-plate substrates and no change compared to PCL controls was observed.

**
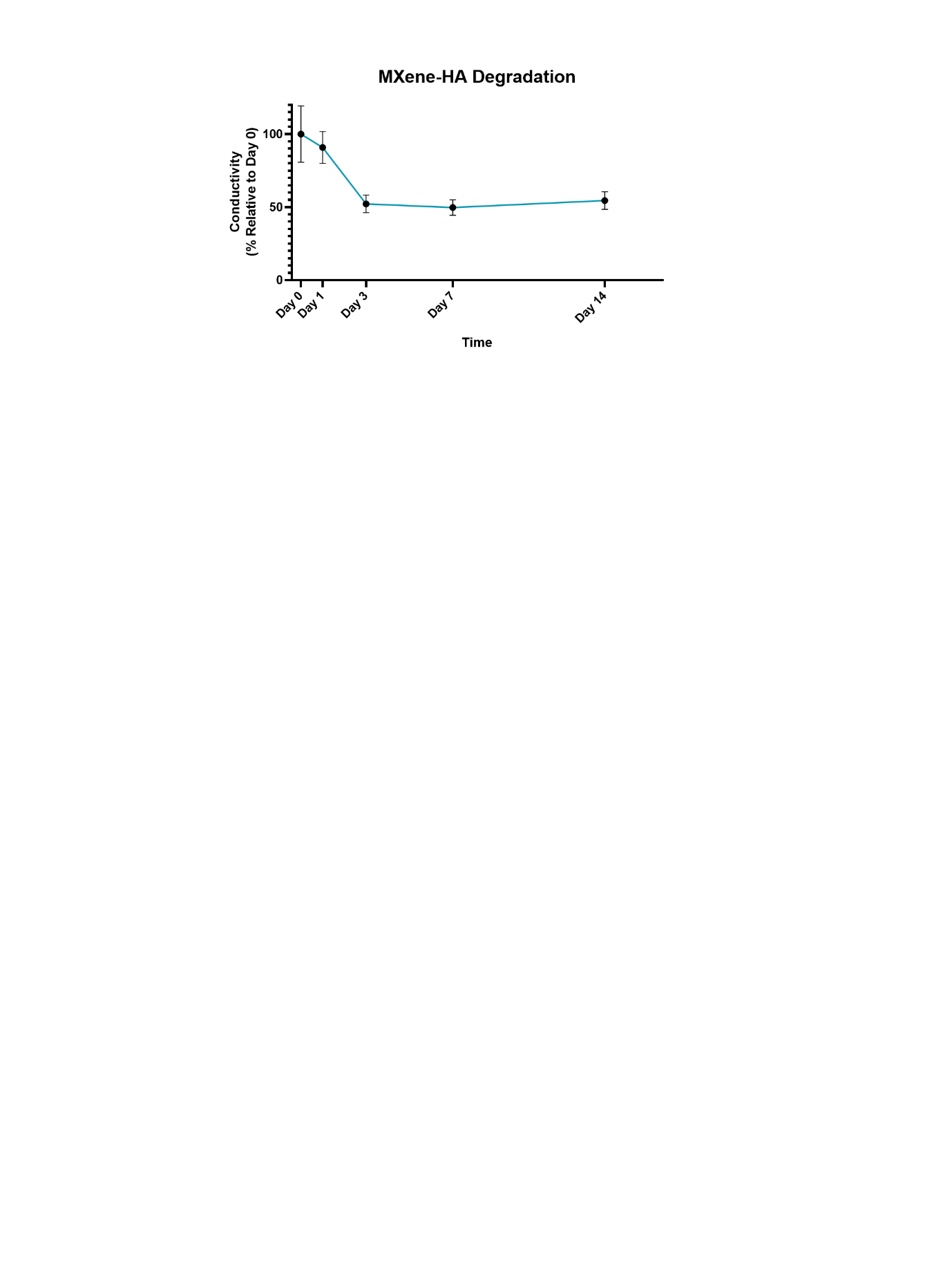
**

**Supplementary Figure S3. Degradation Characteristics.** Medium density MXene-ECM scaffolds incubated in PBS over 14 days. The MXene-ECM scaffolds exhibit an approximate 50% drop in conductivity over 3 days before stabilizing over the two week period. This degradation behaviour is most likely due to separation of the MXene flakes due to swelling and infiltration of water molecules.


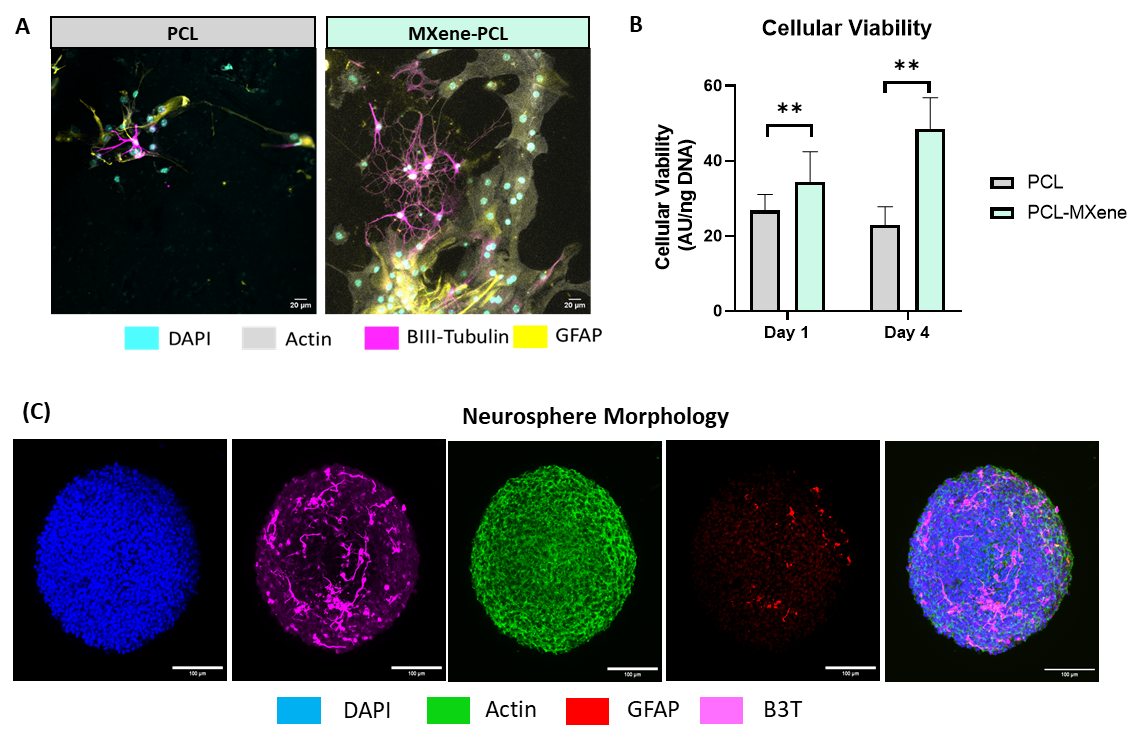


**Supplementary Figure S4. ONSC Biocompatibility and Sphere Morphology.** (A) Immunohistochemical analysis of ONSC cell growth on PCL and MXene-PCL films. Scale bar = 20 µm. (B) Analysis of metabolic activity and DNA content indicates that ONSCs exhibit improved viability on MXene-PCL films compared to PCL controls after 4 days of culture. Scale bar = 100 µm. (C) Neurospheres exhibited a spherical morphology following 10 days of culture, prior to seeding on the scaffold and limited expression of neuronal or glial markers. Scale bar = 100 µm
